# Disorder-specific cognitive profiles in major depressive disorder and generalized anxiety disorder

**DOI:** 10.1186/1471-244X-14-96

**Published:** 2014-04-01

**Authors:** Sanne M Hendriks, Carmilla MM Licht, Jan Spijker, Aartjan TF Beekman, Florian Hardeveld, Ron de Graaf, Brenda WJH Penninx

**Affiliations:** 1 Department of Psychiatry, Pro Persona Mental Health Care, Willy Brandtlaan 20, Ede 6717 RR, The Netherlands; 2 Department of Psychiatry, VU University Amsterdam, A.J. Ernststraat 887, Amsterdam 1081 HL, The Netherlands; 3 Pro Persona Mental Health Care, Radboud University Nijmegen, Reinier Postlaan 6, 6525 GC Nijmegen, The Netherlands; 4 Netherlands Institute of Mental Health and Addiction, Da Costakade 45, Utrecht 3521 VS, The Netherlands; 5 Department of Psychiatry, Leiden University Medical Center, Albinusdreef 2, Leiden 2333 ZA, The Netherlands; 6 Department of Psychiatry, University Medical Center Groningen, Hanzeplein 1, Groningen 9713 GZ, The Netherlands

**Keywords:** Major depressive disorder, Generalized anxiety disorder, Cognitive profiles, Treatment, Classification

## Abstract

**Background:**

This investigation examines differences in cognitive profiles in subjects with major depressive disorder (MDD) and generalized anxiety disorder (GAD).

**Methods:**

Data were used from subjects with current MDD (n = 655), GAD (n = 107) and comorbid MDD/GAD (n = 266) diagnosis from the Netherlands Study of Depression and Anxiety (NESDA). The Composite Interview Diagnostic Instrument was used to diagnose MDD and GAD. Cognitive profiles were measured using the Leiden Index of Depression Sensitivity, the Anxiety Sensitivity Index, and the Penn State Worry Questionnaire.

**Results:**

Results showed that differences in cognitive profiles between single MDD and single GAD subjects were present: scores on hopelessness/suicidality and rumination were significantly higher in MDD than GAD, whereas anxiety sensitivity for physical concerns and pathological worry were higher in GAD than MDD. The cognitive profile of comorbid MDD/GAD showed more extreme depression cognitions compared to single disorders, and a similar anxiety profile compared to single GAD subjects.

**Conclusions:**

Despite the commonalities in cognitive profiles in MDD and GAD, there are differences suggesting that MDD and GAD have disorder-specific cognitive profiles. Findings of this investigation give support for models like the cognitive content-specificity model and the tripartite model and could provide useful handles for treatment focus.

## Background

Major depressive disorder (MDD) and generalized anxiety disorder (GAD) are highly comorbid disorders [1-3]. According to DSM-IV-TR, diagnostic criteria overlap with regard to symptoms like irritability, trouble with concentration, sleeping problems, restlessness, and fatigue [4]. Research in which the underlying latent structure of common psychiatric diagnoses was analysed suggested that GAD belongs to the same dimension as MDD and not to the anxiety disorder dimension [5,6]. There are indications that MDD and GAD share the same genetic risk factors [7,8]. Furthermore, there is overlap in treatment, including use of antidepressants and psychological interventions.

Despite the commonalities between MDD and GAD there are also distinguishing factors. Some research, for instance, gave indications that there are differences in cognitive profiles [9-12]. A number of models have been developed to explain these differences. These include the cognitive content-specificity model [9] and the tripartite model [10]. The cognitive content-specificity model postulates that depressed individuals and anxious individuals differ in their maladaptive thought content. Depressive thought content reflects themes like negative evaluations of the self, the world, and the future, [13,14] whereas anxious thought content reflects only future-oriented concerns with an anticipation of physical or psychological threats and an inability to cope [15,16]. In the tripartite model of anxiety and depression, negative affect is proposed to be associated with both anxiety and depression, whereas lack of positive affect is associated with depression and physiological hyper-arousal is associated with anxiety [10,17,18]. In a revised version of the tripartite model each disorder contains a general, a specific, and a unique component [19]. The general component refers to a factor that anxiety and depressive disorders have in common, the specific component is shared with certain disorders but not all, and the unique component is a characteristic of a particular disorder differentiating it from all the others. Many studies have investigated aspects of the cognitive content-specificity model and the tripartite model [11,20-24]. Previous research showed that the constructs of the cognitive content-specificity model and the tripartite model are meaningfully correlated, and that the integration of the models may better discriminate between anxiety (high negative affect and anxiety cognitions) and depression (high negative affect, low positive affect, and depression cognitions) than either model alone [25-27].

So overall there are suggestions for an overlapping shared latent structure behind GAD and MDD as well as for distinct underlying cognitive vulnerabilities as described in e.g. the tripartite and cognitive content-specificity models. Research on depression and anxiety cognitions across MDD and GAD shows inconsistent findings. A meta-analysis of Naragon-Gainey [28] showed that anxiety cognitions were significantly higher in individuals with GAD compared to individuals with MDD. GAD had a strong association with anxiety sensitivity (tendency to fear anxiety-related sensations) and was related to physical (fear of physical sensations of anxiety), cognitive (fear of mental incapacitation or cognitive dyscontrol), and social concerns (fear of public observation of anxiety). Depression was moderately associated with anxiety sensitivity and was specifically related to cognitive concerns. However, another meta-analysis, [20] in which the cognitive content-specificity hypothesis was evaluated, showed that depression cognitions (mainly measured by the cognitions checklist, i.e. thoughts of loss and failure) are specific to depressive symptomatology, anxiety cognitions (mainly measured by the cognitions checklist, i.e. thoughts of harm and danger) show no such specificity and are associated with both depressive and anxious symptomatology in a similar way. These findings were generally inconsistent with the cognitive content-specificity hypothesis. Also Yook et al. [29] found no significant differences between MDD and GAD in anxiety symptoms and pathological worry, which is a major symptom of GAD (American Psychiatric Association, 1994). Furthermore, Beck et al. (2001) [25] found that worry is correlated with high negative affect and both are shared in depression and anxiety. Their results gave also support that hopelessness is correlated with low positive affect, and is a unique component of depression.

A better understanding of cognitive profiles in MDD and GAD is important for making accurate diagnostic decisions, and would indicate whether treatment approaches developed for MDD might be relevant for GAD and vice versa. Furthermore, it might highlight effective points of intervention for comorbid anxiety and depression symptoms. Little is known about differences and commonalities in cognitive profiles between comorbid MDD/GAD (having both a MDD and a GAD diagnosis) and MDD and GAD, but research indicated that a comorbid MDD/GAD diagnosis shows more severe cognitive problems than a single MDD or GAD diagnosis [30].

Consequently, the extent to which underlying cognitive profiles across GAD and MDD are similar or distinct remains rather unclear. The present study will examine differences in cognitive profiles in three cognitive constructs: cognitive reactivity to sad mood, anxiety sensitivity and pathological worry. Cognitive reactivity to sad mood is defined as the extent to which dysfunctional schemas are activated when mood decreases, [31] and has mainly been investigated in relation to depression as compared to anxiety or GAD. Anxiety sensitivity refers to the fear of anxiety symptoms because of beliefs about their perceived harmful physical, social, or cognitive consequences, [32] and has been linked to all anxiety disorders as well as depression [33-36]. Pathological worry is defined as an unwanted, uncontrollable, aversive cognitive activity associated with negative thoughts and emotional discomfort, [37] and is thought to share the same underlying cognitive process as rumination [38]. There are indications that there is no exclusive relationship between pathological worry and GAD but that pathological worry is also involved in other anxiety disorders and in depression [39,40]. However, not many studies have directly compared MDD and GAD cases in terms of cognitive profiles within one study, or suffered from small sample sizes or lack of consideration of comorbidity of GAD and MDD, which is often present. The present study is set up to do so. We will compare cognitive profiles in rather large groups of subjects with MDD and GAD using data of the Netherlands Study of Depression and Anxiety (NESDA). Furthermore, we will examine to what extent the cognitive profile of subjects with comorbid MDD/GAD is more comparable to that of subjects with single MDD or single GAD.

## Methods

### Study sample

The Netherlands Study of Depression and Anxiety (NESDA) is a naturalistic cohort study to examine the long-term course and consequences of depressive and anxiety disorders. A detailed description of the design and sampling is provided elsewhere [41]. In short, 2981 subjects aged 18 through 65 years were included. The research protocol was approved by the Institutional review boards of the participating institutes (VU University Medical Center, University Medical Center Groningen and Leiden University Medical Center), and all respondents provided written informed consent. The sample consists of subjects with a current or lifetime diagnosis of depression and/or anxiety, and healthy controls. Recruitment took place in the general population, in general practices and in mental health care organisations. Subjects were excluded when they had a primary diagnosis of psychotic, obsessive compulsive, bipolar or severe addiction disorder. Baseline measurements took place between September 2004 and February 2007 and included an assessment of demographic and personal characteristics, a standardized diagnostic psychiatric interview, and a medical assessment.

The Composite Interview Diagnostic Instrument (CIDI version 2.1) [42] was used to diagnose mental disorders, including MDD and GAD. For the present study, we included subjects with 6-month MDD, GAD, and comorbid MDD/GAD diagnoses. In total 1028 subjects were included: 655 with MDD, 107 with GAD and 266 with both MDD and GAD. The mean age of the study sample was 41.6 years, 66.4% was female, and the mean of years of attained education was 11.9 years.

### Cognitive constructs

The Leiden Index of Depression Sensitivity (LEIDS-R), the Anxiety Sensitivity Index (ASI) and the Penn State Worry Questionnaire (PSWQ) were used to examine cognitive profiles in MDD and GAD.

The Leiden Index of Depression Sensitivity (LEIDS-R) [43-45] is a self-report measure of cognitive reactivity to sad mood. The LEIDS-R has 34 items scored on a 5-point Likert scale ranging from “0 = not at all” to “4 = very strongly” and covers six subscales. Subjects were asked to indicate whether and how their thinking patterns change when they experience mild dysphoria. The names of the subscales and sample items are: Hopelessness/Suicidality (5 items, for example: ‘When I feel sad, I feel more hopeless about everything’); Acceptance/Coping (5 items, for example: ‘When I am sad, I feel more like myself’); Aggression (6 items, for example: ‘When I feel down, I lose my temper more easily’); Control/Perfectionism (6 items, for example: ‘When I am in a sad mood, I become more bothered by perfectionism’); Risk Avoidance (6 items, for example: ‘When I feel down, I take fewer risks’); Rumination (6 items, for example: ‘When I feel sad, I spend more time thinking about the possible causes of my moods’). In several studies the LEIDS-R has been found to be sensitive to depression history and they support the validity and the reliability of the LEIDS-R as a measure of depression vulnerability [43-50]. The Cronbach’s α for the LEIDS-R subscales was 0.82 for the subscale Hopelessness/Suicidality, 0.56 for the subscale Acceptance/Coping, 0.79 for the subscale Aggression, 0.61 for the subscale Control/Perfectionism, 0.67 for the subscale Risk Avoidance and 0.70 for the subscale Rumination. Overall, this suggests that the internal reliability of the subscale Acceptance/Coping is not very good, but that of all other subscales are acceptable to good.

Anxiety Sensitivity Index (ASI) [22] is a 16-item questionnaire in which subjects indicate on a 5-point Likert scale (0 = very little to 4 = very much) the degree to which they fear anxiety symptoms (for example: ‘It scares me when my heart beats fast’). Although the ASI originally was postulated to be a unidimensional construct, factor analyses revealed a multidimensional structure. Previous research on NESDA [12] showed that to maintain good internal consistency factors were combined to form 2 factors: a physical concerns factor and a social-cognitive concerns factor. Items 7 and 13 were left out as both items showed very low loadings on each of the 2 factors and removal would improve the internal consistency. The Cronbach’s α for the physical concerns and social-cognitive concerns was 0.88 and 0.79, respectively.

The Penn State Worry Questionnaire (PSWQ) [51] is originally a 16-item inventory designed to assess pathological worry and to capture the generality, excessiveness and uncontrollability characteristics of pathological worry (for example: ‘Once I start worrying, I cannot stop’). Subjects respond using a scale from 1 (not at all typical of me) to 5 (very typical of me). Factor analyses in previous research showed that a two-factor solution provides a better fit [52,53]. Factor 1 (Worry Engagement) demonstrated higher internal consistency and significantly stronger correlations compared to the total PSWQ score and factor 2 (Absence of Worry). For this investigation, we used the questionnaire that contained the 11 items of Worry Engagement. The Cronbach’s α for the PSWQ scale in the current study was 0.92.

### Covariates

Covariates included age, gender, years of education attained, current (6-month recency) comorbid social phobia and comorbid panic disorder.

### Statistical analyses

Statistical analyses were conducted with SPSS Version 20.0 (SPSS Inc, Chicago, Illinois). Chi-square statistics for categorical and analyses of variance for continuous variables were used to compare characteristics across MDD, GAD and comorbid MDD/GAD. Logistic regression analyses determined whether cognitive vulnerability differed significantly between MDD and GAD and whether comorbid MDD/GAD differed from the single disorders. Analyses were corrected for covariates age, gender, years of education attained, current comorbid social phobia, and current comorbid panic disorder. Effect sizes were measured by calculating Cohen’s d [54].

## Results

The baseline characteristics of the respondents are summarized in Table 1. The prevalence of comorbid social phobia and panic disorder was highest in comorbid MDD/GAD (46.2% and 42.9%, respectively) and did differ significantly from MDD.

**Table 1:** Comparison of baseline characteristics between MDD, GAD and comorbid MDD/GAD

	**MDD n = 655**	**GAD n = 107**	**MDD/GAD n = 266**	p*
Sociodemographics				
Age (mean yrs, SD)	41.4 (12.3)	42.0 (12.6)	42.2 (11.7)	.61
Sex, % female	67.3%	60.7%	66.5%	.41
Education (mean yrs, SD)	12.0 (3.2)	11.8 (3.4)	11.6 (3.4)	.41
Comorbidity				
Social phobia	28.2%	37.4%	46.2%	<.001^b^
Panic disorder	31.3%	33.6%	42.9%	.004 ^b^

Note. MDD = major depressive disorder, GAD = generalized anxiety disorder, MDD/GAD = comorbid major depressive disorder and generalized anxiety disorder.

*p-value based on chi-square statistics for categorical variables and analyses of variances for continuous variables (p < .05).

^b^MDD vs MDD/GAD.

Table 2 shows the cognitive profiles for subjects with MDD, GAD, and comorbid MDD/GAD. Hopelessness/suicidality and rumination were significantly higher in MDD compared to GAD (p = .004, Cohen’s d = 0.32 and p = .002, Cohen’s d = 0.30, respectively). Physical concerns were significantly higher in GAD compared to MDD (p < .001, Cohen’s d = 0.32). Pathological worry was significantly higher in GAD compared to MDD (p = .01, Cohen’s d = 0.29). Depression sensitivity and pathological worry were higher in the comorbid MDD/GAD group compared to MDD and GAD but physical concerns were significantly higher in GAD (p = .002, Cohen’s d = 0.24).

**Table 2:** Psychological cognitions of MDD, GAD and comorbid MDD/GAD*

	**MDD n = 655**	**GAD n = 107**	**MDD/GAD n = 266**	p**
Depression sensitivity (LEIDS-R)				
Hopelessness/suicidality	7.2 (4.8)	5.7 (4.5)	8.6 (5.3)	<.001^a,b,c^
Acceptance/coping	1.8 (2.3)	1.7 (2.1)	2.1 (2.4)	.26
Aggression	6.1 (4.8)	5.4 (4.2)	7.1 (4.8)	.01^c^
Control/perfectionism	6.5 (3.9)	6.5 (3.9)	7.3 (3.9)	.10^b^
Risk aversion	10.7 (4.3)	10.3 (4.4)	12.0 (4.6)	.004^b,c^
Rumination	11.7 (4.4)	10.3 (4.8)	13.3 (4.5)	<.001^a,b,c^
Anxiety sensitivity (ASI)				
Physical concerns	7.7 (6.5)	10.7 (7.3)	9.1 (6.1)	<.001^a,c^
Social-cognitive concerns	7.8 (4.5)	8.1 (4.3)	9.5 (4.7)	.01^b,c^
Worry (PSWQ)	36.5 (9.9)	39.2 (8.6)	42.6 (8.5)	<.001^a,b,c^

Note. MDD = major depressive disorder, GAD = generalized anxiety disorder, MDD/GAD = comorbid major depressive disorder and generalized anxiety disorder.

*analyses corrected for covariates age, sex, education, comorbid social phobia and comorbid panic disorder.

**p-value based on analyses of variances (p < .05).

^a^MDD vs GAD.

^b^MDD vs MDD/GAD.

^c^GAD vs MDD/GAD.

Logistic regression analysis was used to determine whether cognitive vulnerability differed significantly between MDD, GAD, and comorbid MDD/GAD (Tables 3 and 4). A model was analysed with the subscales of the LEIDS-R, the ASI, and the PSWQ entered separately (univariable) and entered simultaneously (multivariable). When MDD and GAD were compared (Table 3), hopelessness/suicidality and rumination determined MDD, whereas physical concerns and pathological worry were characteristic for GAD. When cognitive constructs were entered in a multivariable model, all of these results remained significant indicating that these cognitive characteristics were independent of each other.

**Table 3:** Uni- and multivariable associations between cognitive profiles and single MDD (reference) versus single GAD*

	**MDD versus GAD subscales**	
	**Univariable**	**Multivariable**
	**OR (95% CI)**	**OR (95% CI)**
Depression sensitivity (LEIDS-R)**		
Hopelessness/suicidality	1.54 (1.21-1.97)‡	1.47 (1.05-2.04)‡
Acceptance/coping	1.10 (0.88-1.36)	1.08 (0.85-1.37)
Aggression	1.25 (1.00-1.57)†	1.02 (0.77-1.36)
Control/perfectionism	1.04 (0.84-1.28)	0.86 (0.66-1.13)
Risk aversion	1.18 (0.95-1.46)	0.94 (0.69-1.28)
Rumination	1.49 (1.20-1.85)‡	1.78 (1.28-2.46)‡
Anxiety sensitivity (ASI)**		
Physical concerns	0.47 (0.33-0.67)‡	0.42 (0.28-0.65)‡
Social-cognitive concerns	1.02 (0.66-1.57)	1.31 (0.73-2.36)
Pathological worry (PSWQ)**	0.73 (0.55-0.95)‡	0.52 (0.37-0.74)‡

Note. MDD = major depressive disorder, GAD = generalized anxiety disorder.

*based on logistic regression analyses corrected for covariates age, sex, education, comorbid social phobia and comorbid panic disorder.

**all items analysed per SD increase.

†p < .10; ‡p < .05.

**Table 4:** Uni- and multivariable associations between cognitive profiles and single MDD (reference) versus comorbid MDD/GAD, and single GAD (reference) versus comorbid MDD/GAD*

	**MDD versus MDD/GAD subscales**		**GAD versus MDD/GAD subscales**	
	**Univariable**	**Multivariable**	**Univariable**	**Multivariable**
	**OR (95% CI)**	**OR (95% CI)**	**OR (95% CI)**	**OR (95% CI)**
Depression sensitivity (LEIDS-R)**				
Hopelessness/suicidality	0.83 (0.72-0.95)‡	1.03 (0.86-1.25)	0.57 (0.44-0.73)‡	0.70 (0.50-0.97)‡
Acceptance/coping	0.92 (0.80-1.05)	0.95 (0.82-1.11)	0.84 (0.66-1.07)	0.75 (0.55-1.03)†
Aggression	0.88 (0.76-1.01)†	1.03 (0.87-1.22)	0.67 (0.51-0.87)‡	0.91 (0.66-1.25)
Control/perfectionism	0.87 (0.75-1.00)†	1.03 (0.85-1.23)	0.83 (0.66-1.05)	1.21 (0.88-1.66)
Risk aversion	0.82 (0.71-0.95)‡	1.05 (0.86-1.29)	0.72 (0.58-0.91)‡	1.24 (0.89-1.73)
Rumination	0.75 (0.64-0.87)‡	0.93 (0.75-1.17)	0.52 (0.41-0.67)‡	0.57 (0.40-0.82)‡
Anxiety sensitivity (ASI)**				
Physical concerns	0.83 (0.64-1.10)	1.19 (0.86-1.64)	1.92 (1.26-2.94)‡	4.03 (2.24-7.24)‡
Social-cognitive concerns	0.63 (0.47-0.85)‡	0.91 (0.62-1.33)	0.59 (0.36-0.96)‡	0.47 (0.23-0.95)‡
Pathological worry (PSWQ)**	0.46 (0.37-0.56)‡	0.45 (0.35-0.57)‡	0.61 (0.44-0.84)‡	0.88 (0.59-1.31)

Note. MDD = major depressive disorder, GAD = generalized anxiety disorder, MDD/GAD = comorbid major depressive disorder and generalized anxiety disorder.

*based on logistic regression analyses corrected for covariates age, sex, education, comorbid social phobia and comorbid panic disorder.

**all items analysed per SD increase.

†p < .10; ‡p < .05.

For MDD versus comorbid MDD/GAD (Table 4) hopelessness/suicidality, risk aversion, rumination, social-cognitive concerns, and pathological worry determined comorbid MDD/GAD. When the items were entered in a multivariable model, only pathological worry remained significant indicating higher levels in case of comorbid MDD/GAD.

When comparing GAD and comorbid MDD/GAD (Table 4), hopelessness/suicidality, aggression, risk aversion and rumination, and social-cognitive concerns were characteristics of comorbid MDD/GAD, whereas higher physical concerns determined GAD. When a multivariable model was analysed, higher scores of hopelessness/suicidality, rumination, and social-cognitive concerns determined comorbid MDD/GAD, whereas higher physical concerns determined GAD.

## Discussion

The aim of this investigation was to examine cognitive profiles in MDD and GAD, and to examine whether cognitive profiles differentiate between these disorders. Furthermore, we wanted to investigate if the cognitive profile of comorbid MDD/GAD is more comparable with MDD or with GAD. The results showed that there are differences in depression and anxiety cognitions in MDD and GAD, although differences showed moderate effect sizes. Furthermore, most subscales showed similar results for MDD and GAD. Previous research is inconsistent about these findings. For example, in a meta-analysis Beck & Perkins [20] showed that depression cognitions are relative unique to depression but anxiety cognitions were shared in depression and anxiety, which is in contrast with our findings. Differences can be explained by the use of different cognitive constructs (for example the Cognitions Checklist that was not included in NESDA), different sample sizes, and the fact that investigations did not always take place in clinical settings. Other research, however, demonstrated that MDD and GAD indeed have disorder-specific cognitive profiles [11]. Despite the commonalities in cognitive profiles in MDD and GAD, we also found differences that seem to be characteristic for the disorders. We found that hopelessness/suicidality and rumination are more common in MDD whereas physical concerns and pathological worry are more common in GAD. Our findings are in line with previous research, [13,21,25,28,29] and seem to fit in models like the cognitive content-specificity model and the tripartite model of anxiety and depression.

Our results showed that rumination is more prominent in MDD whereas worry is more prominent in GAD. Although our findings are much in line with previous studies, [55-57] the assertion that worry only relates to anxiety and rumination only to depression has been challenged. Worry has been found to be associated with depression, and rumination with anxiety [37,58-60]. There is a general consensus that rumination and worry share some degree of overlap. However some researchers argue how similar rumination and worry are. Rumination has been found significantly correlated to worry [60,61]. Furthermore, Segerstrom et al. (2000) [61] found that both rumination and worry are forms of repetitive thinking. Watkins et al. (2005) [62] examined differences and similarities between rumination and worry and found very few differences. One difference in their study was the temporal content of the intrusive thoughts. Worry thoughts were more concerned with the future and less concerned with the past than ruminative thoughts which was in line with previous research [56]. So it seems that rumination is oriented towards the past and is more strongly associated with depression, while worry is oriented towards the future and is more strongly associated with GAD.

We found higher scores on hopelessness/suicidality in MDD compared to GAD. Previous research reported that hopelessness is a stronger indicator of depression than anxiety, and may distinguish individuals with MDD from those with GAD [15,21,25]. Some recent studies point at the role of intolerance of uncertainty, [21,29,63-65] which can be defined as a cognitive bias that affects how a person perceives, interprets, and responds to uncertain situations on a cognitive emotional and behavioural level [66]. It has been suggested that intolerance of uncertainty is of influence on the different paths leading to MDD or GAD. The point at which individuals become certain about both the absence of a positive future and the presence of a negative future leads to hopelessness about the future, and this leads to increased symptoms of depression (e.g. suicidality) [21,67,68]. However, Sargalska et al. (2011) showed that only certainty about the absence of a positive future (and not the presence of a negative future) predicts suicidal ideation, even after adjusting for hopelessness and symptoms of depression [69].

Another finding in our study was that physical concerns are more prominent in GAD compared to MDD, but cognitive and social concerns do not differ between GAD and MDD. Anxiety sensitivity refers to the fear of anxiety symptoms because of beliefs about their perceived harmful physical, social, or cognitive consequences [32]. Most of earlier research focused on the relation between anxiety sensitivity and panic disorder, and since the early 1990s research has expanded to include other internalizing disorders. There is some evidence suggesting that the anxiety sensitivity dimensions correspond to specific anxiety disorders. Physical concerns are more strongly associated with panic disorder, social concerns are more strongly associated with social phobia, and cognitive concerns are more strongly associated with GAD [33-35,70]. Previous research indicated that anxiety sensitivity is not only associated with anxiety but also with depression, [33,35,36] raising the question whether anxiety sensitivity is specific for anxiety disorders or for distress in general. In a meta-analysis, Olantunji and Wolitzky-Taylor (2009) [71] showed that individuals with anxiety disorders reported greater overall anxiety sensitivity than those with mood disorders. However, significant effect sizes were only found for panic disorder. It has been argued that physical and social concerns are more specific for anxiety, whereas cognitive concerns are more specific for depression [35]. Another meta-analysis [28] showed that GAD had a strong association with anxiety sensitivity and is related to physical, cognitive, and social concerns, whereas depression was moderately associated with anxiety sensitivity and is specifically related to cognitive concerns. Relating to our results, there seems to be overlap in cognitive and social concerns in MDD and GAD but the physical component seems to be more specific for anxiety.

The cognitive profile of comorbid MDD/GAD showed more extreme depression cognitions than both single disorders, and a comparable anxiety profile compared to GAD. These results may suggest that comorbidity between MDD and GAD denotes a more severe illness since previous research showed that severity is strongly related to comorbidity [2]. As mentioned before, depression and anxiety have notably high rates of comorbidity. Cognitive constructs may shed some light on the issue of comorbidity but also other factors seem important. For example, previous research showed a systematic link between personality traits and psychopathology [72]. Possibly, personality traits may serve as vulnerability factors or may be the underlying cause of a disorder. For instance, neuroticism/negative emotionality (stress reactivity and a tendency to experience negative emotions) has shown to be elevated in both depressive and anxiety disorders and consequently could contribute to comorbidity among them [73].

This study has several strengths. The large sample size, including well diagnosed subjects with affective and anxiety disorders allows in-depth analyses of the cognitive profiles of subjects with MDD and GAD. Subjects were recruited in both primary care and specialized mental health care increasing the generalizability of our findings to standard clinical settings.

Limitations of this study should be acknowledged as well. First, the cross-sectional study design precludes any causal interpretations regarding the cognitive profiles in MDD and GAD, and longitudinal studies are necessary to address such questions. Second, GAD was not the only anxiety disorder present and we did not exclude subjects with other anxiety disorders. However, analyses were corrected for comorbid panic disorder and social phobia and comorbidity patterns were not different across single MDD and single GAD groups. Subjects were excluded when they had a clinically primary diagnosis of severe other psychiatric disorders such as psychotic, obsessive compulsive, bipolar or severe addiction disorder. However, some other conditions may have been present which were not further assessed, such as posttraumatic stress disorder, specific phobia, personality disorder or eating disorder. Consequently, we could not examine the full range of comorbidity among GAD and MDD in more detail. Third, the group sizes differed substantially: the GAD group was smaller compared to the MDD group, which indicates that statistical significance was harder to obtain for the single GAD group comparisons. However, when examining the results of the study, this does not seem to have affected the conclusions of the paper. Finally, a selection has been made among the available measurements of depression and anxiety cognitions. It would have been interesting to include intolerance of uncertainty, which is discussed broadly because of the possible high influence on the different paths leading to MDD and GAD [21,29].

## Conclusion

The classification of MDD and GAD was under scrutiny in the process leading to DSM-5 and there has been a number of calls to reclassify GAD in the same category as MDD (‘the distress disorders’). Early GAD definitions suffered considerably from low reliability and poor validity. However, worry became the essential feature of GAD in DSM-III-R [74]. DSM-IV further clarified the diagnosis by focusing on physical symptoms around chronic levels of tension and removing symptoms that more likely reflect acute autonomic arousal. These changes increased the diagnostic reliability and helped to distinguish GAD from other anxiety and mood disorders and demonstrated unique mechanisms and patterns of impairment. This is an important reason why MDD and GAD are still represented as distinct disorders in DSM-5.

The results of this study demonstrate that subjects with MDD and GAD have disorder-specific cognitive profiles, despite commonalities in other aspects. Specific depression cognitions, such as rumination, hopelessness, and suicidality, are more prominent in MDD whereas specific anxiety cognitions, such as physical concerns and pathological worry, are more prominent in GAD. These findings suggest that both MDD and GAD have specific features and may support a separate classification of MDD and GAD. Furthermore, the cognitive profile of comorbid MDD/GAD showed more extreme depression cognitions than both single disorders, and a comparable anxiety profile compared to GAD. The findings of this investigation give support for models like the cognitive content-specificity model and the tripartite model and could provide useful handles for treatment focus.

## Competing interests

The authors declare that they have no competing interests.

## Authors’ contributions

SH participated in the design of the study, performed and interpreted the statistical analyses and wrote the manuscript. CL interpreted the statistical analyses and has critically revised the manuscript. JS, AB, FH and RdG have critically revised the manuscript. BP is the principal investigator of the NESDA study, and participated in the design of the study and revising the manuscript. All authors read and approved the final manuscript.

## Pre-publication history

The pre-publication history for this paper can be accessed here:

http://www.biomedcentral.com/1471-244X/14/96/prepub

## References

[B1] Kessler RC Nelson CB McGonagle KA Liu J Swartz M Blazer DG Comorbidity of DSM-III-R major depressive disorder in the general population: results from the US National Comorbidity Survey Br J Psychiatry Suppl 1996 17 30 8864145

[B2] Kessler RC Chiu WT Demler O Walters EE Prevalence, severity, and comorbidity of twelve-month DSM-IV disorders in the National Comorbidity Survey Replication (NCS-R) Arch Gen Psychiatry 2005 62 617 627 10.1001/archpsyc.62.6.617 15939839 PMC2847357

[B3] Kessler RC Gruber M Hettema JM Hwang I Sampson N Yonkers KA Comorbid major depression and generalized anxiety disorders in the National Comorbidity Survey follow-up Psychol Med 2008 38 365 374 18047766 10.1017/S0033291707002012 PMC2745899

[B4] American Psychiatric Association Diagnostic and Statistical Manual of Mental Disorders 1994 4 Washington, DC: American Psychiatric Association

[B5] Krueger RF The structure of common mental disorders Arch Gen Psychiatry 1999 56 921 926 10.1001/archpsyc.56.10.921 10530634

[B6] Vollebergh WAM Iedema J Bijl RV de Graaf R Smit F Ormel J The structure and stability of common mental disorders Arch Gen Psychiatry 2001 58 597 603 10.1001/archpsyc.58.6.597 11386990

[B7] Kendler KS Major depression and generalized anxiety disorder. Same genes, (partly) different environments – revisited Br J Psychiatry 1996 168 Suppl 30 68 75 8864151

[B8] Kendler KS Gardner CO Gatz M Pedersen NL The sources of co-morbidity between major depression and generalized anxiety disorder in a Swedish national twin sample Psychol Med 2007 37 453 462 10.1017/S0033291706009135 17121688

[B9] Clark DA Beck AT Kendall PC, Watson D Cognitive theory and therapy of anxiety and depression Anxiety and Depression: Distinctive and Overlapping Features 1989 San Diego: Academic Press 379 411

[B10] Clark LA Watson D Tripartite model of anxiety and depression: psychometric evidence and taxonomic implications J Abnorm Psychol 1991 100 316 336 1918611 10.1037//0021-843x.100.3.316

[B11] Ghahramanlou-Holloway M Wenzel A Lou K Beck AT Differentiating cognitive content between depressed and anxious outpatients Cogn Behav Ther 2007 36 170 178 10.1080/16506070701374256 17852174

[B12] Drost J Van der Does AJW Antypa N Zitman FG Van Dyck R Spinhoven P General, specific and unique cognitive factors involved in anxiety and depressive disorders Cogn Ther Resin press 10.1007/s10608-011-9401-z PMC3490077 23144515

[B13] Clark DA Beck AT Brown G Cognitive mediation in general psychiatric outpatients: a test of the content-specificity hypothesis J Pers Soc Psychol 1989 56 958 964 2746459 10.1037//0022-3514.56.6.958

[B14] Clark DA Beck AT Stewart B Cognitive specificity and positive–negative affectivity: complementary or contradictory views on anxiety and depression? J Abnorm Psychol 1990 99 148 155 2348008 10.1037//0021-843x.99.2.148

[B15] Beck AT Clark DA Anxiety and depression: an information processing perspective Anxiety Res 1988 1 23 36 10.1080/10615808808248218

[B16] Tellegen A Tuma AH, Maser JD Anxiety and anxiety disorders Structures of Mood and Personality and Their Relevance to Assessing Anxiety with and Emphasis on Self-Report 1985 Hillsdale, NJ: Erlbaum 681 706

[B17] Clark LA Watson D Mineka S Temperament, personality, and the mood and anxiety disorders J Abnorm Psychol 1994 103 103 116 8040472

[B18] Watson D Tellegen A Toward a consensual structure of mood Psychol Bull 1985 98 219 235 3901060 10.1037//0033-2909.98.2.219

[B19] Mineka S Watson D Clark LA Comorbidity of anxiety and unipolar mood disorders Annu Rev Psychol 1998 49 377 412 10.1146/annurev.psych.49.1.377 9496627

[B20] Beck R Perkins TS Cognitive content-specificity for anxiety and depression: a meta-analysis Cogn Ther Res 2001 25 651 663 10.1023/A:1012911104891

[B21] Miranda R Fontes M Marroquín B Cognitive content-specificity in future expectancies: role of hopelessness and intolerance of uncertainty in depression and GAD symptoms Behav Res Ther 2008 46 1151 1159 10.1016/j.brat.2008.05.009 18687420

[B22] Norton PJ Sexton KA Walker JR Norton GR Hierarchical model of vulnerabilities for anxiety: replication and extension with a clinical sample Cogn Behav Ther 2005 34 50 63 10.1080/16506070410005401 15844688

[B23] Norton PJ Mehta PD Hierarchical model of vulnerabilities for emotional disorders Cogn Behav Ther 2007 36 240 254 10.1080/16506070701628065 18049949

[B24] Sexton KA Norton PJ Walker JR Norton GR Hierarchical model of generalized and specific vulnerabilities in anxiety Cogn Behav Ther 2003 32 82 94 10.1080/16506070302321 16291539

[B25] Beck R Perkins TS Holder R Robbins M Gray M Allison SH The cognitive and emotional phenomenology of depression and anxiety: are worry and hopelessness the cognitive correlates of NA and PA? Cogn Ther Res 2001 25 829 838 10.1023/A:1012983726272

[B26] Beck R Benedict B Winkler A Depression and anxiety: integrating the tripartite and cognitive content-specificity assessment models Psychopathol Behav Assess 2003 25 251 256 10.1023/A:1025899029040

[B27] Jolly JB Dyck MJ Kramer TA Wherry JN Integration of positive and negative affectivity and cognitive content-specificity: improved discrimination of anxious and depressive symptoms J Abnorm Psychol 1994 103 544 552 7930054 10.1037//0021-843x.103.3.544

[B28] Naragon-Gainey K Meta-analysis of the relations of anxiety sensitivity to the depressive and anxiety disorders Psychol Bull 2010 136 128 150 20063929 10.1037/a0018055

[B29] Yook K Kim K Suh SY Lee KS Intolerance of uncertainty, worry, and rumination in major depressive disorder and generalized anxiety disorder J Anxiety Disord 2010 24 623 628 10.1016/j.janxdis.2010.04.003 20439149

[B30] Dupuy JB Ladouceur R Cognitive processes of generalized anxiety disorder in comorbid generalized anxiety disorder and major depressive disorder J Anxiety Disord 2008 22 505 514 10.1016/j.janxdis.2007.05.010 17600670

[B31] Miranda J Gross JJ Persons JB Hahn J Mood matters: negative mood induction activates dysfunctional attitudes in women vulnerable to depression Cogn Ther Res 1998 22 363 376 10.1023/A:1018709212986

[B32] Reiss S Peterson RA Gursky DM McNally RJ Anxiety sensitivity, anxiety frequency and the prediction of fearfulness Behav Res Ther 1986 24 1 8 10.1016/0005-7967(86)90143-9 3947307

[B33] Rector NA Szacun-Shimizu K Leybman M Anxiety sensitivity within the anxiety disorders: disorder-specific sensitivities and depression comorbidity Behav Res Ther 2007 45 1967 1975 10.1016/j.brat.2006.09.017 17084380

[B34] Rodriguez BF Bruce SE Pagano ME Spencer MA Keller MB Factor structure and stability of the anxiety sensitivity index in a longitudinal study of anxiety disorder patients Behav Res Ther 2004 42 79 91 10.1016/S0005-7967(03)00074-3 14744525 PMC3272759

[B35] Taylor S Koch WJ Woody S McLean P Anxiety sensitivity and depression: how are they related? J Abnorm Psychol 1996 105 474 479 8772020 10.1037//0021-843x.105.3.474

[B36] Zinbarg RE Brown TA Barlow DH Rapee RM Anxiety sensitivity, panic, and depressed mood: a reanalysis teasing apart the contributions of the two levels in the hierarchical structure of the anxiety sensitivity index J Abnorm Psychol 2001 110 372 377 11502080 10.1037//0021-843x.110.3.372

[B37] Borkovec TD Davey GCL, Tallis F The nature, functions and origins of worry Worrying: Perspectives on Theory, Assessment and Treatment 1994 Chichester, England: John Wiley

[B38] Watkins ER Constructive and unconstructive repetitive thought Psychol Bull 2008 134 163 206 18298268 10.1037/0033-2909.134.2.163 PMC2672052

[B39] Starcevic V Pathological worry in major depression: a preliminary report Behav Res Ther 1995 33 55 56 10.1016/0005-7967(93)E0028-4 7872937

[B40] Starcevic V Berle D Milicevic D Hannan A Lamplugh C Eslick GD Pathological worry, anxiety disorders and the impact of co-occurrence with depressive and other anxiety disorders J Anxiety Disord 2007 21 1016 1027 10.1016/j.janxdis.2006.10.015 17270391

[B41] Penninx BWJH Beekman ATF Smit JH Zitman FG Nolen WA Spinhoven PH Cuijpers P de Jong PJ van Marwijk HWJ Assendelft WJJ van der Meer K Verhaak P Wensing M de Graaf R Hoogendijk WJ Ormel J van Dyk R The Netherlands Study of Depression and Anxiety (NESDA): rationale, objectives and methods Int J Methods Psychiatr Res 2008 17 121 140 10.1002/mpr.256 18763692 PMC6878352

[B42] Wittchen HU Reliability and validity studies of the WHO-Composite International Diagnosic Interview (CIDI): a critical review J Psychiatr Res 1994 28 57 84 10.1016/0022-3956(94)90036-1 8064641

[B43] Van der Does W Cognitive reactivity to sad mood: structure and validity of a new measure Behav Res Ther 2002 40 105 120 10.1016/S0005-7967(00)00111-X 11762423

[B44] Antypa N Van der Does AJW Penninx BWJH Cognitive reactivity: investigation of a potentially treatable marker of suicide risk in depression J Affect Disord 2010 122 46 52 10.1016/j.jad.2009.06.013 19584020

[B45] Booij L Van der Does AJW Cognitive and serotonergic vulnerability to depression: convergent findings J Abnorm Psychol 2007 116 86 94 17324019 10.1037/0021-843X.116.1.86

[B46] Van der Does W Thought suppression and cognitive vulnerability to depression Br J Clin Psychol 2005 44 1 14 10.1348/014466504x19442 15826340

[B47] Firk C Markus CR Mood and cortisol responses following tryptophanrich hydrolyzed protein and acute stress in healthy subjects with high and low cognitive reactivity to depression Clin Nutr 2009 28 266 271 10.1016/j.clnu.2009.03.002 19345451

[B48] Merens W Booij L Markus R Zitman FG Onkenhout W Van der Does AJW The effects of a diet enriched with α-lactalbumin on mood and cortisol response in unmedicated recovered depressed subjects and controls Br J Nutr 2005 94 415 422 10.1079/BJN20051492 16176613

[B49] Moulds ML Kandris E Williams AD Lang T Yap C Hoffmeister K An investigation of the relationship between cognitive reactivity and rumination Behav Ther 2008 39 65 71 10.1016/j.beth.2007.05.001 18328871

[B50] Williams JMG Van der Does AJW Barnhofer T Crane C Segal ZS Cognitive reactivity, suicidal ideation and future influence: preliminary investigation of a differential activation theory of hopelessness/suicidality Cogn Ther Res 2008 321 83 104

[B51] Meyer TJ Miller ML Metzger RL Borkovec TD Development and validation of the Penn State Worry Questionnaire Behav Res Ther 1990 28 487 495 10.1016/0005-7967(90)90135-6 2076086

[B52] Fresco DM Heimberg RG Mennin DS Turk CL Confirmatory factor analysis of the Penn State Worry Questionnaire Behav Res Ther 2002 40 313 323 10.1016/S0005-7967(00)00113-3 11863241

[B53] Brown TA Confirmatory factor analysis of the Penn State Worry Questionnaire: multiple factors or method effects? Behav Res Ther 2003 41 1411 1426 10.1016/S0005-7967(03)00059-7 14583411

[B54] Cohen J Statistical Power Analysis for the Behavioral Sciences 1988 2 Hillsdale, NJ: Lawrence Erlbaum Associates

[B55] Becker ES Goodwin R Hölting C Hoyer J Margraf J Content of worry in the community: what do people with generalized anxiety disorder or other disorders worry about? J Nerv Ment Dis 2003 191 688 691 10.1097/01.nmd.0000092198.20420.fc 14555873

[B56] Chelminsky I Zimmerman M Pathological worry in depressed and anxious patients J Anxiety Disord 2003 17 533 546 10.1016/S0887-6185(02)00246-3 12941364

[B57] Papageorgiou C Wells A Process and meta-cognitive dimensions of depressive and anxious thoughts and relationships with emotional intensity Clin Psychol Psychother 1999 6 156 162 10.1002/(SICI)1099-0879(199905)6:2<156::AID-CPP196>3.0.CO;2-A

[B58] Hong RY Worry and rumination: differential associations with anxious and depressive symptoms and coping behavior Behav Res Ther 2007 45 277 290 10.1016/j.brat.2006.03.006 16635479

[B59] Nolen-Hoeksema S The role of rumination in depressive disorders and mixed anxiety/depressive symptoms J Abnorm Psychol 2000 3 504 511 11016119

[B60] Fresco DM Frankel AN Mennin DS Turk CL Heimberg RG Distinct and overlapping features of rumination and worry: the relationship of cognitive production to negative affective states Cogn Ther Res 2002 26 179 188 10.1023/A:1014517718949

[B61] Segerstrom SC Tsa JCI Alden LE Craske MG Worry and rumination: repetitive thought as a concomitant and predictor of negative mood Cogn Ther Res 2000 24 671 688 10.1023/A:1005587311498

[B62] Watkins E Moulds M Mackintosh B Comparisons between rumination and worry in a non-clinical population Behav Res Ther 2005 43 1577 1585 10.1016/j.brat.2004.11.008 16239152

[B63] Buhr K Dugas MJ Investigating the construct validity of intolerance of uncertainty and its unique relationship with worry J Anxiety Disord 2006 20 222 236 10.1016/j.janxdis.2004.12.004 16464706

[B64] Dugas MJ Schwartz A Francis K Intolerance of uncertainty, worry, and depression Cogn Ther Res 2004 28 835 842 10.1007/s10608-004-0669-0

[B65] Miranda R Mennin DS Depression, generalized anxiety disorder, and certainty in pessimistic predictions about the future Cogn Ther Res 2007 31 71 82 10.1007/s10608-006-9063-4

[B66] Buhr K Dugas MJ The intolerance of uncertainty scale: psychometric properties of the English version Behav Res Ther 2002 40 931 945 10.1016/S0005-7967(01)00092-4 12186356

[B67] Abramson LY Metalsky GI Alloy LB Hopelessness depression: a theory-based subtype of depression Psychol Rev 1989 96 358 372

[B68] Andersen SM The inevitability of future suffering: the role of depressive predictive certainty in depression Soc Cogn 1990 8 203 228 10.1521/soco.1990.8.2.203

[B69] Sargalska J Miranda R Marroquin B Being certain about an absence of the positive: specificity in relation to hopelessness and suicidal ideation Int J Cogn Ther 2011 4 104 116 10.1521/ijct.2011.4.1.104

[B70] Zinbarg RE Barlow DH Brown TA Hierarchical structure and general factor saturation of the Anxiety Sensitivity Index: Evidence and implications Psychol Assess 1997 9 277 284

[B71] Olatunji BO Wolitzky-Taylor KB Anxiety sensitivity and the anxiety disorders: a meta-analytic review and synthesis Psychol Bull 2009 135 974 999 19883144 10.1037/a0017428

[B72] Krueger RF McGue M Iacono WG The higher-order structure of common DSM mental disorders: internalization, externalization, and their connections to personality Pers Individ Dif 2001 30 1245 1259 10.1016/S0191-8869(00)00106-9

[B73] Watson D Gamez W Simms LJ Basic dimensions of temperament and their relation to anxiety and depression: a symptom-based perspective J Res Pers 2005 39 46 66 10.1016/j.jrp.2004.09.006

[B74] American Psychiatric Association Diagnostic and Statistical Manual of Mental Disorders 1987 3 Washington, DC: American Psychiatric Association

